# Cardiac phenotype in children with X-linked ichthyosis (*STS*-sEDD): findings from a prospective cohort

**DOI:** 10.1007/s00431-026-07488-z

**Published:** 2026-10-09

**Authors:** Katia Henostroza Inga, Andrea Greco, Estefanía Martínez-Barrios, José Cruzalegui, Nuria Díez-Escuté, Sonia Ehrenberg Archs, Laura Valero Martín, Beatriz Mínguez, Marta Bové Guri, Georgia Sarquella-Brugada, Eulalia Baselga Torres

**Affiliations:** 1 Department of Dermatology, Hospital Sant Joan de Déu Barcelona, Barcelona, Spain; 2 Pediatric Arrhythmias, Genetic Cardiology and Sudden Death, Institut de Recerca Sant Joan de Déu (IRSJD), Barcelona, Spain; 3 Arrhythmia, Inherited Cardiac Diseases and Sudden Death Unit, Department of Cardiology, Hospital Sant Joan de Déu Barcelona, Barcelona, Spain; 4 Department of Pediatrics, School of Medicine, University of Barcelona, Barcelona, Spain; 5 Department of Pediatrics, Hospital Sant Joan de Déu Barcelona, Barcelona, Spain; 6 Department of Gastroenterology, Hospital Sant Joan de Déu Barcelona, Barcelona, Spain; 7 Department of Ophthalmology, Hospital Sant Joan de Déu Barcelona, Barcelona, Spain; 8 Department of Medical Sciences, School of Medicine, University of Girona, Girona, Spain

**Keywords:** X-linked ichthyosis, *STS* deletion, Pediatric cohort, Cardiac evaluation, Syndromic epidermal differentiation disorder

## Abstract

X-linked ichthyosis is caused by pathogenic variants or deletions involving *STS* at Xp22.31. Because some deletions include contiguous genes and cardiac rhythm abnormalities have been reported, we characterized the cardiac phenotype of children with genetically confirmed steroid sulfatase-related syndromic epidermal differentiation disorder (*STS*-sEDD). This prospective, observational, single-centre study included 14 boys aged 2–16 years. Assessment comprised clinical history and examination, 12-lead electrocardiography, echocardiography, exercise testing when age-appropriate, and 5-day ambulatory electrocardiographic monitoring. Genetic findings were reviewed, and 11 obligate-carrier mothers also underwent cardiological assessment. Nine patients (64%) were asymptomatic and five (36%) reported mild, nonspecific symptoms. Minor electrocardiographic findings in 11 patients were considered age-appropriate variants. All hearts were structurally normal except for one previously known, haemodynamically compensated partial anomalous pulmonary venous return. Exercise testing in 12 patients identified one isolated premature ventricular contraction; 5-day monitoring in 13 detected no clinically significant arrhythmia. Twelve patients had contiguous-gene deletions and two had pathogenic *STS* sequence variants, with no apparent association between deletion extent and cardiac findings. Maternal echocardiograms were normal; one mother had isolated atrial and ventricular ectopy during exercise without clinically significant arrhythmia.

*Conclusion*: Clinically relevant cardiac involvement appeared uncommon in this small pediatric cohort. The sample size and cross-sectional assessment preclude definitive risk estimates; cardiological evaluation should be individualized according to symptoms, cardiovascular findings, and family history.

**Table:** 

**What is Known:** • *X-linked ichthyosis is caused by STS variants or deletions that may involve contiguous genes.* • *Cardiac arrhythmias have been reported with Xp22.31 deletions, but pediatric data are scarce.*
**What is New:** • *No clinically significant arrhythmia was found in 14 boys undergoing multimodal assessment.* • *Deletion extent did not predict cardiac findings; follow-up should reflect symptoms and family history.*

**What is Known:**

• *X-linked ichthyosis is caused by STS variants or deletions that may involve contiguous genes.*

• *Cardiac arrhythmias have been reported with Xp22.31 deletions, but pediatric data are scarce.*

**What is New:**

• *No clinically significant arrhythmia was found in 14 boys undergoing multimodal assessment.*

• *Deletion extent did not predict cardiac findings; follow-up should reflect symptoms and family history.*

## Introduction

**Fig. 1:**
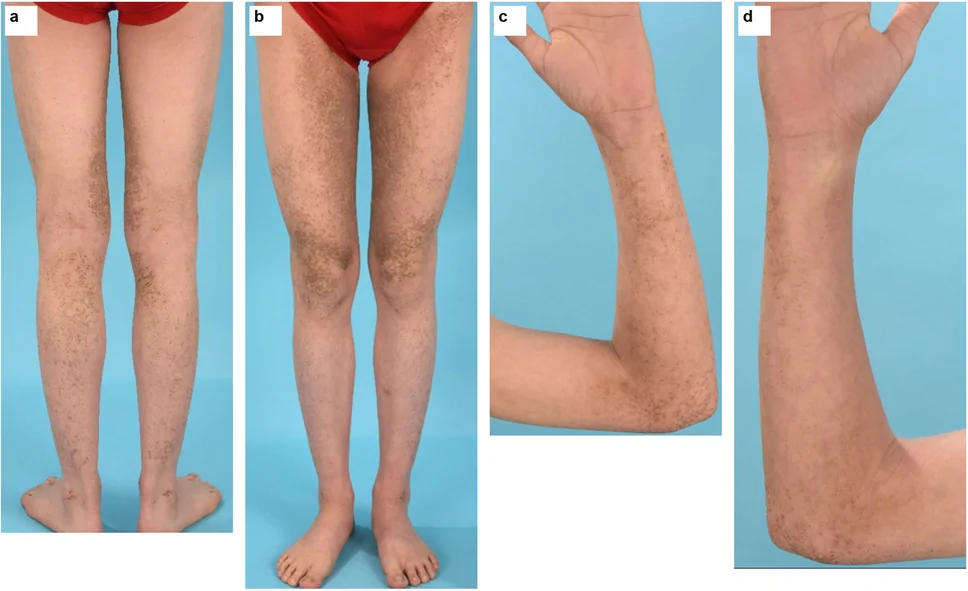
Cutaneous manifestations of X-linked ichthyosis. **a**, **b **Lower limbs showing generalized polygonal, dark-brown scaling. **c**, **d** Upper limbs showing generalized xerosis and scaling on extensor surfaces. *STS*-sEDD, steroid sulfatase-related syndromic epidermal differentiation disorder

X-linked ichthyosis (XLI) is an almost exclusively male disorder characterized by generalized scaling. Typical features include polygonal dark-brown scales on the lower limbs and neck, fine periauricular scaling, and larger rhomboid scales on extensor surfaces (Fig. 1). The face, skin folds, palms, soles, nails, and teeth are usually spared [1]. XLI results from pathogenic variants or deletions involving *STS* at Xp22.31. Steroid sulfatase hydrolyses cholesterol sulfate to free cholesterol in the epidermis; deficiency causes cholesterol sulfate accumulation, impaired corneocyte desquamation, hyperkeratosis, and adherent scales [1].

Historically classified as a non-syndromic ichthyosis, XLI is now included among syndromic epidermal differentiation disorders as *STS*-sEDD [2, 3]. Many recurrent deletions extend beyond *STS* and include neighbouring genes or regulatory elements such as *VCX*, *VCX3A*, *VCX2*, *MIR651*, *MIR4767*, *PUDP*, and *PNPLA4* [4–9]. Variation in deletion extent and effects on neighbouring loci may contribute to heterogeneous extracutaneous manifestations.

Recent reports have raised concern about cardiac rhythm disturbances in *STS*-sEDD, including bradycardia, right bundle branch block, prolonged asystole, ventricular arrhythmia, and atrial fibrillation [4, 5, 8, 9]. However, systematic pediatric cardiological data remain limited. We therefore characterized structural and rhythm findings in a pediatric cohort with genetically confirmed *STS*-sEDD and explored whether symptoms or deletion extent were associated with clinically relevant cardiac abnormalities.

## Materials and methods

### Study design and participants

This prospective, observational, single-centre study evaluated male patients with clinically and molecularly confirmed *STS*-sEDD at the multidisciplinary ichthyosis clinic of Hospital Sant Joan de Deu Barcelona, Spain, between January 2023 and July 2025. Biologically related mothers, who were obligate carriers of the familial *STS* alteration, were also invited to undergo cardiological assessment.

### Cardiological assessment

Pediatric assessment included the following: (1) clinical history and physical examination focused on dizziness, palpitations, chest pain, syncope, and other symptoms suggestive of arrhythmia; (2) standard 12-lead electrocardiography (ECG); (3) transthoracic echocardiography; (4) symptom-limited exercise testing using the standard Bruce treadmill protocol in age-appropriate children; and (5) 5-day ambulatory single-lead ECG monitoring using ePatch (Philips). Clinically significant arrhythmias were defined as sustained supraventricular or ventricular arrhythmia, high-grade atrioventricular block, or another rhythm abnormality considered clinically relevant by the evaluating pediatric cardiologist. Cardiological investigations were performed and interpreted by pediatric cardiologists from the same pediatric cardiology unit according to the standardized study protocol. Depending on clinical scheduling, individual participants could be evaluated by different members of the cardiology team. No formal blinded or duplicate independent adjudication of the cardiac investigations was performed.

### Genetic analysis

Array comparative genomic hybridization used high-density oligonucleotide arrays (qGenomics), and multiplex ligation-dependent probe amplification used the SALSA MLPA P160-C1 kit (MRC-Holland). Clinical exome sequencing used the TruSight One panel (Illumina), with copy-number variant detection by ExomeDepth. Copy-number variants were interpreted according to ACMG/ClinGen recommendations [10], and sequence variants according to ACMG/AMP guidelines [11]. Deleterious sequence variants were confirmed by Sanger sequencing when appropriate. MLPA and/or array-CGH was used to characterize copy-number variants involving *STS* and neighbouring genes within Xp22.31. Deletion extent and involvement of contiguous genes were descriptively compared with cardiological findings.

### Statistical analysis

Because of the exploratory design and small sample, analyses were descriptive. Continuous variables are presented as mean and range, and categorical variables as counts and percentages. No inferential statistical analyses were performed.

### Ethics

The study was conducted in accordance with the Declaration of Helsinki and approved by the Ethics Committee of Hospital Sant Joan de Deu Barcelona (PIC-108-23). Written informed consent was obtained before participation.

## Results

### Participants

**Fig. 2:**
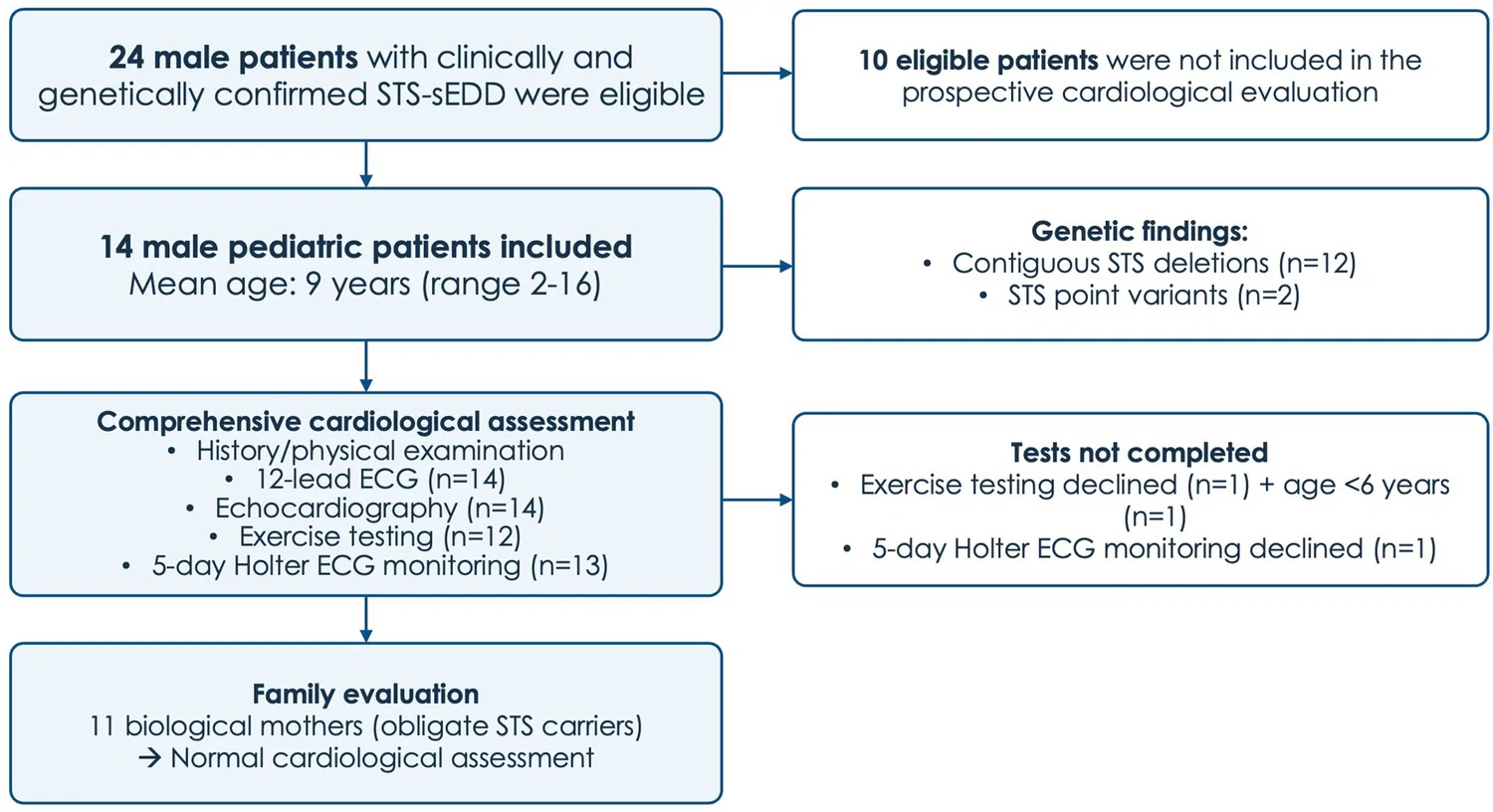
Study flowchart

Twenty-four male patients with clinically and genetically confirmed *STS*-sEDD were eligible. Fourteen underwent prospective cardiological evaluation (mean age 9 years; range 2–16 years), while 10 did not undergo the prospective assessment (Fig. 2).

### Clinical and cardiological findings

Nine patients (64%) were asymptomatic and five (36%) reported mild, nonspecific symptoms: postural dizziness (*n *= 3), occasional dizziness (*n* = 1), or exercise-related chest pain (*n* = 1). None of these symptoms was associated with a clinically relevant cardiac abnormality (Table 1). One patient had a history of an infant heart murmur, and another had a previously diagnosed partial anomalous pulmonary venous return (PAPVR) involving the right pulmonary vein. One patient had a maternal uncle with XLI, dilated cardiomyopathy, and ventricular fibrillation requiring an implantable cardioverter-defibrillator.

**Table 1:** Clinical characteristics, cardiological evaluation, and genetic findings in pediatric patients with *STS*-sEDD (*n* = 14)

Domain	Variable	Patients, *n* (%)	Key findings	Clinical interpretation
Personal and family history	Family history of severe arrhythmia	1 (7%)	Maternal uncle with XLI, DCM, and ventricular fibrillation requiring ICD	Relationship to XLI uncertain
	Infant heart murmur	1 (7%)	Detected during infancy	Isolated finding without functional significance
Clinical characteristics	Postural dizziness	3 (21%)	Mild, intermittent episodes	No associated structural disease or arrhythmia
	Occasional dizziness	1 (7%)	Isolated symptom	No cardiological abnormality identified
	Exercise-related chest pain	1 (7%)	Early repolarization with normal exercise test	Non-ischaemic chest pain
	No cardiac symptoms	9 (64%)	-	Asymptomatic
Electrocardiography	Sinus tachycardia	1 (7%)	Physiological rhythm	Age-appropriate variant
	Sinus bradycardia	2 (14%)	Asymptomatic	Age-appropriate variant
	Sinus arrhythmia	5 (36%)	Respiratory-related variation	Age-appropriate variant
	Ectopic atrial rhythm	1 (7%)	No symptoms	Benign ECG variant
	Early repolarization	1 (7%)	In patient with chest pain	Benign ECG variant
	Incomplete right bundle branch block	1 (7%)	Incidental finding	Common benign variant
	Normal ECG	3 (21%)	-	-
Echocardiography	PAPVR	1 (7%)	Previously known right pulmonary vein anomaly	Only structural cardiac anomaly
	Structurally normal heart with trivial tricuspid regurgitation	8 (57%)	Normal estimated pulmonary pressures	Physiological pediatric finding
Exercise testing (*n* = 12)	Isolated PVC	1/12 (8%)	Single ectopic beat during exercise	Not clinically significant
	Normal	11/12 (92%)	No ischaemia or arrhythmia	Normal functional evaluation
Five-day ambulatory ECG (*n* = 13)	No arrhythmia detected	13/13 (100%)	Normal rhythm monitoring	No clinically relevant arrhythmia
Genetic findings	Contiguous-gene deletion including *STS*	12 (86%)	Three recurrent deletion patterns	Pathogenic copy-number variants
	Pathogenic *STS* sequence variant	2 (14%)	Pathogenic sequence variants	Consistent with *STS*-sEDD
Clinical-genetic correlation	Mild symptoms without cardiac pathology	5 (36%)	Symptoms not associated with structural disease or arrhythmia	Overall reassuring pediatric phenotype

Percentages use the full pediatric cohort (*n* = 14) unless a tested denominator is shown. *DCM* Dilated cardiomyopathy, *ECG* Electrocardiography, *ICD* Implantable cardioverter-defibrillator, *PAPVR* Partial anomalous pulmonary venous return, *PVC* Premature ventricular contraction, *STS-sEDD* Steroid sulfatase-related syndromic epidermal differentiation disorder, *XLI* X-linked ichthyosis

**Fig. 3:**
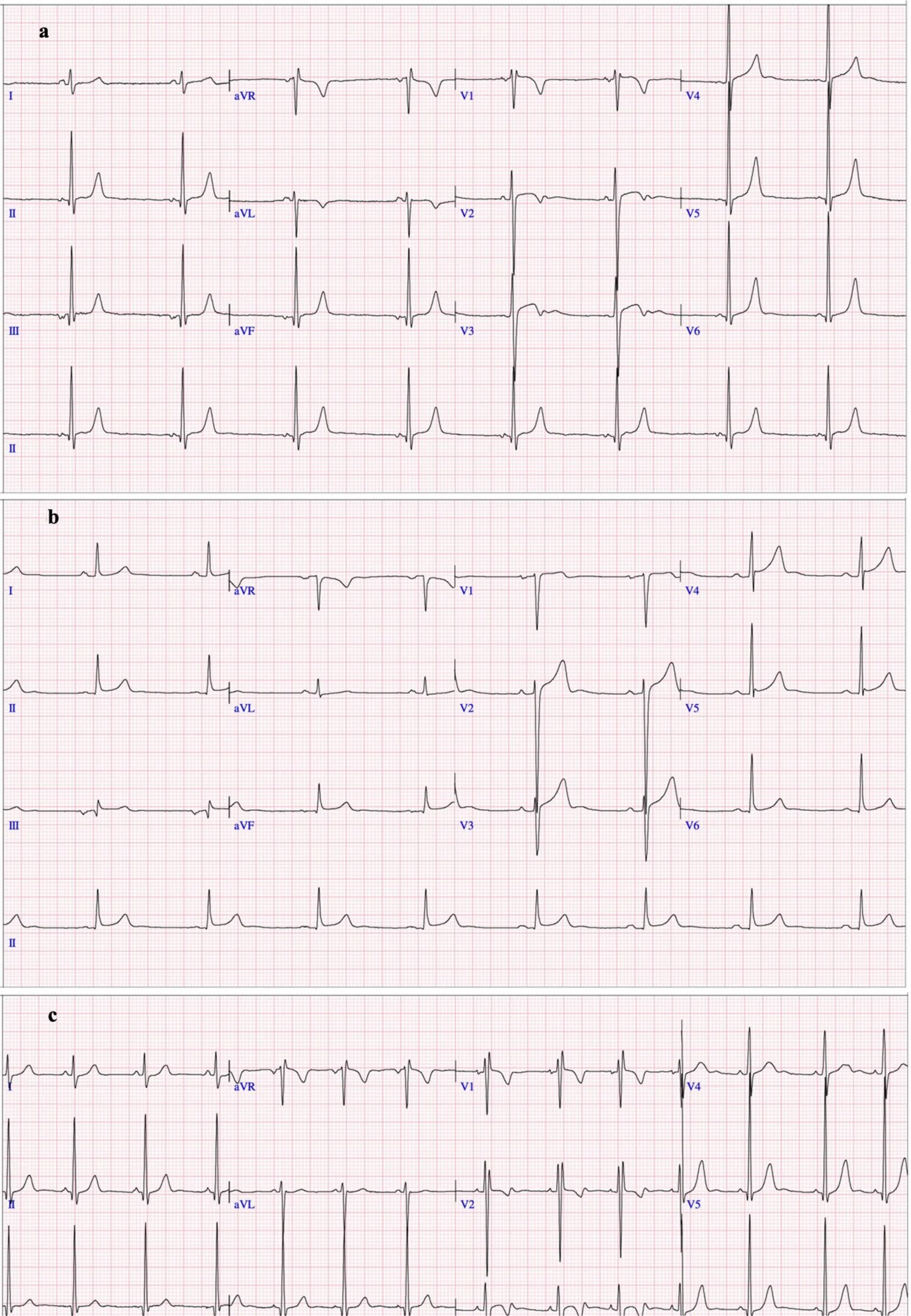
Electrocardiographic findings observed in children with X-linked ichthyosis. **a** Ectopic atrial rhythm. **b** Early repolarization pattern. **c** Incomplete right bundle branch block. ECG, electrocardiogram

Minor ECG variations were observed in 11 patients (79%): sinus tachycardia (*n* = 1), sinus bradycardia (*n* = 2), sinus arrhythmia (*n* = 5), ectopic atrial rhythm (*n* = 1; Fig. 3a), early repolarization (*n* = 1; Fig. 3b), and incomplete right bundle branch block (*n* = 1; Fig. 3c). All were classified as age-related physiological variants rather than pathological conduction abnormalities.

Echocardiography showed structurally normal hearts in all participants except for the patient with previously diagnosed, haemodynamically compensated PAPVR. Trivial tricuspid regurgitation with normal estimated pulmonary artery pressure was present in eight patients and considered physiological. Exercise testing was completed in 12 patients; one patient declined and another was younger than 6 years. One isolated premature ventricular contraction was recorded during exercise and was not considered clinically significant. Five-day ambulatory ECG monitoring was completed in 13 patients; one declined. No clinically significant arrhythmias were detected.

### Family cardiological evaluation

Eleven biological mothers who were obligate carriers underwent cardiological evaluation. Echocardiographic findings were normal in all. One mother (9%) had isolated premature atrial and ventricular contractions during exercise testing, without structural heart disease or clinically significant arrhythmia.

### Genetic findings and genotype–phenotype correlation

**Fig. 4:**
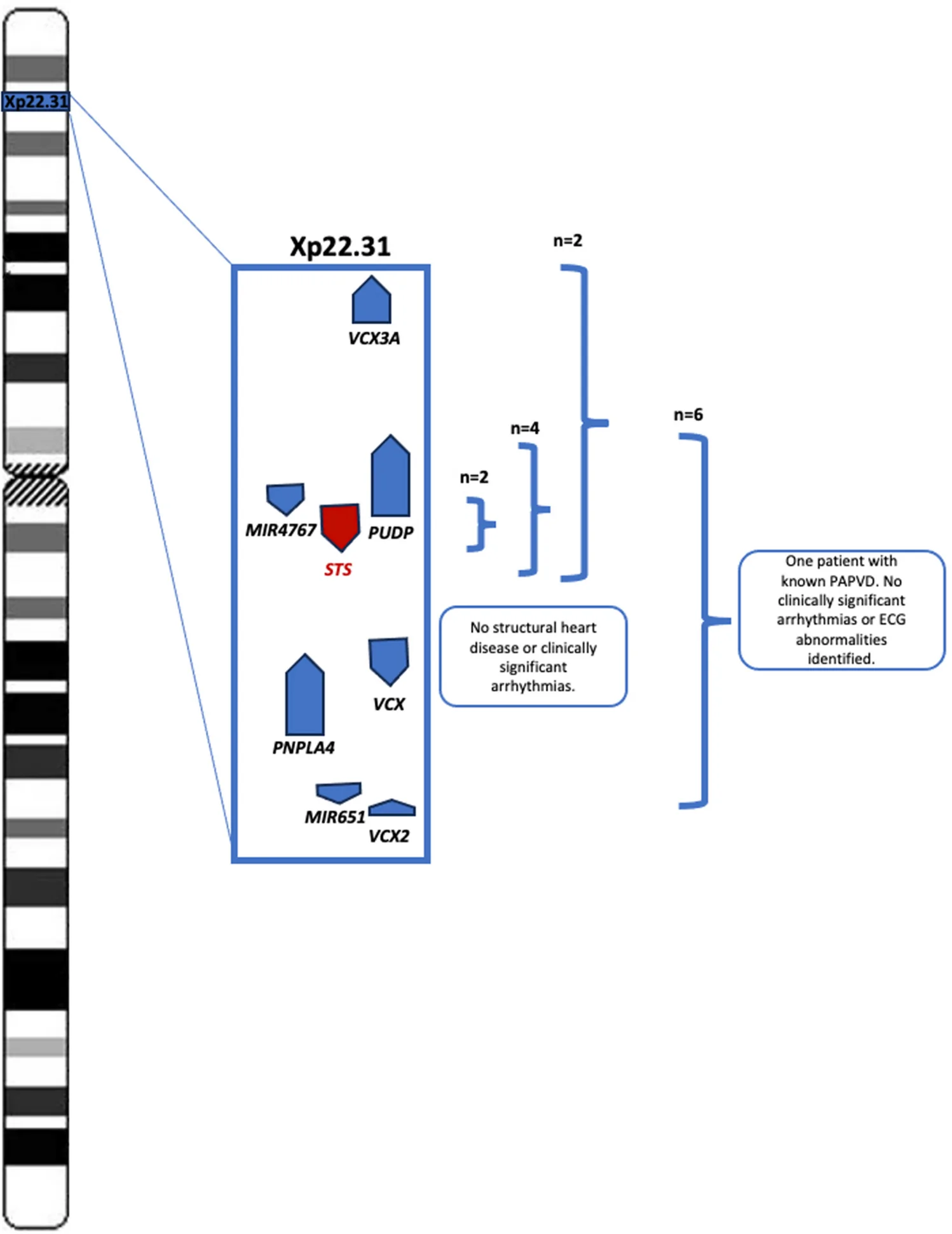
Genomic organization of the Xp22.31 region showing *STS* and contiguous genes affected in the cohort

Twelve patients had contiguous-gene deletions extending beyond *STS*. The most frequent deletion included *PUDP*, *MIR4767*, *STS*, *VCX*, *PNPLA4*, and *MIR651* (*n* = 6; 43%), followed by deletions involving *PUDP*, *MIR4767*, and *STS* (*n* = 4; 29%) and *VCX3A*, *PUDP*, *MIR4767*, and *STS* (*n* = 2; 14%). Two patients had pathogenic *STS* sequence variants (*n* = 2; 14%) (Table 2; Fig. 4). No apparent association was observed between deletion extent or contiguous-gene involvement and structural heart disease, clinically significant arrhythmia, or other abnormal cardiological findings. The patients with PAPVR and with a family history of ventricular fibrillation both carried the largest contiguous deletion, but neither had a clinically significant arrhythmia during the study.

**Table 2:** Genotype–phenotype findings in the pediatric *STS*-sEDD cohort

Genetic finding	*n* (%)	Cardiac findings
*PUDP*, *MIR4767*, *STS*, *VCX*, *PNPLA4*, and *MIR651* contiguous deletion	6 (43%)	One patient with previously known PAPVR; no clinically significant arrhythmia
*PUDP*, *MIR4767*, and *STS* contiguous deletion	4 (29%)	No structural heart disease or clinically significant arrhythmia
*VCX3A*, *PUDP*, *MIR4767*, and *STS* contiguous deletion	2 (14%)	No structural heart disease or clinically significant arrhythmia
Pathogenic *STS* sequence variant	2 (14%)	No structural heart disease or clinically significant arrhythmia
Overall cohort	14 (100%)	No apparent association between deletion extent and clinically relevant cardiac abnormalities

## Discussion

This prospective study provides a standardized cardiological characterization of children with genetically confirmed *STS*-sEDD. Studies of adults with XLI-associated deletions have reported atrial fibrillation, atrial flutter, and other rhythm disturbances, with some data suggesting increased risk among middle-aged men [4, 5, 8, 9]. In contrast, clinical assessment, 12-lead ECG, echocardiography, exercise testing, and prolonged ambulatory ECG monitoring identified no clinically significant arrhythmia or previously unrecognized structural heart disease in this pediatric cohort. Most ECG and echocardiographic observations were age-appropriate variants.

The difference between reassuring pediatric findings and reported adult rhythm abnormalities raises the possibility of age-dependent expression. Steroid sulfatase deficiency alters cholesterol sulfate metabolism and the balance between sulfated and non-sulfated steroid hormones, including dehydroepiandrosterone sulfate, estrogens, and androgens [12–14]. These pathways could influence electrophysiology and structural remodelling over time. Our cross-sectional observations cannot determine whether abnormalities emerge with age; longitudinal follow-up from childhood into adulthood is required.

Most participants carried deletions extending beyond *STS* and involving neighbouring genes or regulatory elements. Potential effects on lipid metabolism, neuronal regulation, mitochondrial function, and epigenetic processes have been proposed [15–19]. We observed no consistent association between deletion extent and clinically relevant cardiac findings, but the small genetic subgroups preclude definitive genotype-phenotype conclusions. Larger deletions should therefore not be regarded as protective or as independently predictive on the basis of these data.

Severe pediatric cardiac manifestations have nevertheless been reported. Dantsev et al. described an adolescent with an Xp22.31 deletion, progressive sinus node dysfunction, severe bradycardia, prolonged asystolic pauses, polymorphic ventricular ectopy, QTc shortening, and recurrent syncope who required pacemaker implantation [5]. Such reports show that clinically important cardiac disease may occur in childhood, although causality cannot be established from an individual case.

Intrafamilial variability was also evident. One child shared a large contiguous deletion with a maternal uncle who had XLI, dilated cardiomyopathy, and ventricular fibrillation requiring an implantable cardioverter-defibrillator, yet the child had a reassuring evaluation. Additional cardiomyopathy- or arrhythmia-related variants, epigenetic influences, environmental exposures, and age may have contributed to the adult relative’s phenotype. This finding supports assessment based on symptoms, family history, and individual cardiovascular findings rather than the *STS* alteration alone.

The assessment of obligate female carriers was exploratory. All maternal echocardiograms were normal, and one mother had isolated atrial and ventricular ectopy during exercise without clinically significant arrhythmia. The small number of women and non-dedicated design do not permit conclusions about cardiac risk in heterozygous carriers.

### Clinical implications

These results are reassuring but should be interpreted cautiously. The data do not support intensive universal cardiac surveillance solely because of *STS*-sEDD in asymptomatic children; however, the study is too small to exclude uncommon events. A baseline cardiological assessment may be considered to identify unrecognized abnormalities and establish a reference. Pediatric cardiology referral should be prioritized for syncope, sustained palpitations, documented arrhythmia, structural abnormalities, or a family history of sudden cardiac death or malignant arrhythmia. Transition to adult care should preserve cardiovascular history and enable reassessment if symptoms or risk factors emerge.

### Strengths and limitations

The principal strength is the standardized evaluation using complementary structural and rhythm tests, including 5-day ambulatory monitoring. Several limitations should be acknowledged. This single-centre study included only 14 evaluated patients, limiting precision, precluding inferential analysis, and reducing the ability to detect rare manifestations or genotype-phenotype associations. Ten of 24 eligible patients did not undergo prospective cardiological evaluation, creating potential selection bias; included and non-included patients were not formally compared.

Not every participant completed each investigation: exercise testing was available in 12 and ambulatory ECG monitoring in 13. Cardiac investigations were interpreted clinically by members of the pediatric cardiology team without formal blinded or duplicate independent adjudication. The assessment was cross-sectional and did not include follow-up through adolescence and adulthood. Accordingly, the absence of clinically significant arrhythmias in this cohort does not exclude rare cardiac events or arrhythmias that may emerge later with age. The maternal evaluation was small and not designed to quantify risk in heterozygous women. Multicentre longitudinal studies with larger cohorts and standardized cardiovascular assessment are needed.

## Conclusion

In this pediatric cohort with genetically confirmed *STS*-sEDD, comprehensive cardiological evaluation identified no clinically significant arrhythmia or previously unrecognized structural heart disease. Clinically relevant cardiac involvement may be uncommon during childhood, but the small sample and cross-sectional design preclude definitive risk estimates. No apparent association between deletion extent and cardiac findings was observed. Evaluation and follow-up should be individualized according to symptoms, cardiovascular findings, and family history, with longitudinal studies needed to define age-related risk.

## Data Availability

The dataset, entitled “Cardiac phenotype in children with X-linked ichthyosis (STS-sEDD),” is held securely at Hospital Sant Joan de Déu Barcelona. De-identified data may be made available by the corresponding author upon reasonable request, subject to approval by the institution and the relevant ethics committee and in accordance with applicable data-protection regulations.

## Abbreviations

ACMG: American College of Medical Genetics and Genomics
ACMG/AMP: American College of Medical Genetics and Genomics/Association for Molecular Pathology
CGH: Comparative genomic hybridization
CNV: Copy-number variant
DCM: Dilated cardiomyopathy
ECG: Electrocardiography/electrocardiogram
ICD: Implantable cardioverter-defibrillator
MLPA: Multiplex ligation-dependent probe amplification
PAPVR: Partial anomalous pulmonary venous return
PVC: Premature ventricular contraction
*STS*-sEDD: Steroid sulfatase-related syndromic epidermal differentiation disorder
XLI: X-linked ichthyosis

## Declarations

### Reporting guideline

The authors completed the STROBE checklist for this observational study.
